# Quantification of postural stability in minimally disabled multiple sclerosis patients by means of dynamic posturography: an observational study

**DOI:** 10.1186/s12984-016-0216-8

**Published:** 2017-01-10

**Authors:** Lucia Grassi, Stefano Rossi, Valeria Studer, Gessica Vasco, Caterina Motta, Fabrizio Patanè, Enrico Castelli, Silvia Rossi, Paolo Cappa

**Affiliations:** 1 Department of Mechanical and Aerospace Engineering, “Sapienza” University of Rome, Rome, Italy; 2 Department of Economics and Management, Industrial Engineering, University of Tuscia, Viterbo, Italy; 3 Dipartimento di Medicina dei Sistemi, Tor Vergata University, Rome, Italy; 4 Department of Neurosciences, Movement Analysis and Robotics Laboratory (MARLab), Neurorehabilitation Unit, IRCCS Bambino Gesù Children’s Hospital, Rome, Italy; 5 School of Mechanical Engineering, “Niccolò Cusano” University, Rome, Italy; 6 Neuroimmunology and Neuromuscular Diseases Unit, Foundation Neurological Institute Carlo Besta, Milan, Italy

**Keywords:** Static posturography, Dynamic posturography, Multiple sclerosis, Equilibrium assessment, Balance control, Cerebellar impairments

## Abstract

**Background:**

Multiple Sclerosis (MS) is a widespread progressive neurologic disease with consequent impairments in daily activities. Disorders of balance are frequent and equilibrium tests are potentially useful to quantify disability and to verify treatment effectiveness. The fair sensitivity of the widely used not-perturbed tests to detect balance disturbances in MS patients have prompted the development of mechatronic systems capable to impose known equilibrium perturbations, in order to challenge the balance control and, consequently, to better assess the level of impairment. We sought to clarify whether the proposed perturbed-test is capable to discriminate healthy subjects from patients with MS, even in mild or in the absence of clinically evident balance disturbances.

**Methods:**

We assessed balance performances of 17 adults with MS and 13 age-matched healthy controls (HC) using both perturbed (PT) and not-perturbed (NPT) postural tests by means of a 3 Degree Of Freedom (DOF) rotational mechatronic platform. Participants stood barefoot on the platform in standing position and their center of pressure (CoP) was gathered by using a pressure matrix. Each trial lasted 30 s and was carried out with and without visual stimuli. Several postural indices were computed for each trial. Correlations between postural indices and clinical scales were analyzed.

**Results:**

No significant differences were found between groups for all indices when subjects performed NPTs. Conversely, significant differences in postural indices between MS and HC emerged during PTs. Additionally, PTs revealed significant differences between patients without any cerebellar impairment (cerebellar EDSS subscore equal to 0) and HC. The discrimination capability of PTs was confirmed by the ROC analysis. No significant change of the selected metrics occurred in HC when NPTs were performed with eyes closed, while indices presented a significant worsening in MS subjects.

**Conclusions:**

Not-perturbed tests showed lower sensitivity than perturbed ones in the identification of equilibrium impairments in minimally disabled MS patients. However, not-perturbed tests allow to better evaluate the influence of visual flow disturbances on balance control in MS. In conclusion, our findings proved that the use of the novel tests based on a 3DOF mechatronic device represents an effective tool to investigate early balance disturbances in MS.

## Background

Multiple Sclerosis (MS) is a chronic disorder of the Central Nervous System (CNS), characterized by inflammation, demyelination and neurodegenerative features, and is one of the principal causes of neurological disability in young adults [1]. Visual, somatosensory, and vestibular systems, on which postural control relies, are frequently involved in the disease, leading to balance and coordination disturbances [2] and to an increased risk of falls [3]. An early identification of the patients who are at higher risk of accidental falls is essential to define the need of specific interventions and to optimize rehabilitation programs.

The most common clinical scales for equilibrium assessment are unable to detect minor balance deficits in mildly disabled patients [4]. Several instrumented balance tests have therefore been proposed to study equilibrium control in healthy subjects [5, 6], to assess the severity of balance disorders in patients with neurological diseases [7, 8], and to verify the effectiveness of the selected clinical treatments [9, 10]. Balance tests can be grouped depending on the absence or presence of movements of the support base on which the subjects stand during the posture test; the first condition is generally addressed to Not-perturbed Test (NPT) and the latter one as Perturbed Test (PT).

NPTs are mainly based on the analysis of the ground reaction forces and the Centre of Pressure (CoP) trajectory generated by the body sway [11]; subjects are asked to maintain a quiet standing posture on a support, either rigid or with a foam applied on [2] and by instructing participants to stay with eyes open (EO) or closed (EC) [12]. Specifically, the outputs of NPTs have been used to evaluate recovery or progression of the pathological condition in patients with cerebellar or labyrinthine lesions [13] and with Parkinson disease [8]. Moreover, NPTs have been performed to subjects with MS to challenge their postural abilities, to assess their balance impairment [14, 15], to evaluate the capability of NPTs in falls prediction [16], and to assess the outcomes of rehabilitation programs [2]. Promising results have been reported about sensitivity of NPTs in detecting balance disturbances in minimally disabled MS patients [17, 18]; moreover, a greater sensitivity of NPTs in evaluating risk of falls was found in MS subjects in comparison with questionnaires and standard clinical tests [15, 19], with greater specificity for lower EDSS scores [15, 19]; however, these findings are not conclusive, due to the great variability of testing conditions, which limit the comparison of results.

PTs can be categorized in two subgroups according to the balance perturbations provided when subjects are standing on a platform; specifically, the perturbation can be either self-generated by a movement of the subject, such as reaching tasks or load release [20–22], or imposed by an external source. As regards self-generated perturbations, as an example, Karst et al. [21] found differences of CoP displacements between subjects with MS and healthy control group during reaching and leaning tasks. As regards mechanical perturbation, instead, the most widespread test is the Sensory Organization Test (SOT, NeuroCom, Natus Medical Incorporated, US) that can impose rotational perturbations in the anteroposterior direction to the subjects. SOT has been widely used in healthy subjects [23, 24], in subjects with vestibular dysfunctions [25, 26] and in MS patients, to elucidate specific postural responses related to MS severity [27, 28] and to investigate the benefits of the rehabilitation treatments [10]. Di Fabio et al. [25] used SOT to detect vestibular deficits, finding a correlation between balance performances and vestibular impairment, as well as Schwab et al. [26]. Fjeldstad et al. [27] tested SM subjects by using SOT and showed significant differences on postural stability between MS and control groups and a good correlation between the standard Berg Balance Scale BBS and the SOT score. Finally, Fjeldstad et al. [28] found that the balance parameters of subjects with MS were significantly worse than the healthy controls, indicating a greater postural instability despite the low disability. Although the SOT is the most widespread test, recently Peterson et al. [29] studied the effects of linear perturbations in subjects with MS by means of a robotic platform moving in forward and backward directions. They found that the measurement of backward compensatory stepping can be useful to identify postural dysfunction in subjects with MS.

The intrinsic limit of the SOT and of the other proposed robotic platforms is that the systems can supply only simple perturbations of the base support that are constrained along the anteroposterior direction. This limitation does not allow performing studies focused on the evaluation of postural control strategies in presence of complex perturbations that act also in mediolateral direction. Even if anteroposterior perturbations are able to find correlation between equilibrium and chronic diseases, they do not allow evaluating the presence of asymmetry in the control strategy selected by subjects in order to maintain the equilibrium. To overcome this limitation, complex perturbations acting also in mediolateral direction are needed to deeply analyze postural control strategies. The quantification of postural responses to lateral rotations was also one of the best predictors of future falls [30]. Therefore, the quantification of postural responses to complex perturbations can be useful to predict balance in functional activities and to investigate the presence of a preferential direction of movement in MS disease. To the best of our knowledge, no studies have been up to now conducted to: (i) propose and verify PTs based on complex perturbations of the base support and (ii) provide objective measures of the postural control capabilities of subjects with MS.

In this work, we proposed a novel PT based on an in-house developed 3-DOF mechatronic device [31–34] to evaluate the postural control strategies in MS patients both in anteroposterior and in mediolateral directions. We decided to conduct the quantitative assessment on a metrics based on the Center of Pressure (CoP) time histories. In our working hypothesis we sought to clarify whether the proposed novel PT protocol is capable to discriminate healthy subjects from patients with MS, even in mild or in the absence of clinically evident balance disturbances.

## Methods

### Subjects

A cohort of seventeen patients (8 M, 9 F; aged: 43.4 ± 9.0 years) with a diagnosis of MS [35], was assessed at the Movement Analysis and Robotics Laboratory of the “Bambino Gesù” Children’s Hospital in Rome. Only patients with cerebellar EDSS subscore ≤ 2 and no history of recurrent accidental falls related to imbalance were selected to test the sensitivity of PT protocol in detecting equilibrium impairments even in the case of patient with mild cerebellar impairment with no clinical evidence of balance dysfunction. Patients could not be enrolled if they relapsed in the 60 days preceding inclusion. Other exclusion criteria were: the need for an orthosis for stance control of the foot, ankle, and/or knee; motor impairment with symptoms that limited participation in study activities (piramidal EDSS subscore > 2); the receipt of dalfampridine for the treatment of MS symptoms to avoid possible positive or negative influences of this drug on motor performance. In fact, dalfampridine is reported to improve motor performance of lower limbs but also to induce dizziness and vertigos as possible side effects [36, 37]. Demographic and clinical details were derived from medical records and shown in Table 1. MS subjects underwent clinical evaluation with EDSS assessment the same day of posturography evaluation.

**Table 1:** Demographic and clinical characteristics of subjects

	HC	MS	*p*	S0	S1	*p*
Number	13	17		10	7	
Age [years]	39.4 ± 7.2	43.4 ± 9.0	0.20	41.4 ± 10.6	46.4 ± 5.3	0.27
Sex (M/F)	9/4	8/9	0.28	5/5	¾	0.99
Disease duration [years]	−	10.1 ± 7.4	−	9.4 ± 9.2	11.1 ± 4.1	0.65
EDSS	−	2.3 ± 1.6	−	1.5 ± 1.4	3.3 ± 1.4	0.02

*M* male, *F* female, *HC* healthy control, *MS* multiple sclerosis, *S0* MS patients without sensory involvement, *S1* *MS* patients with sensory involvement, *EDSS* expanded disability status scale

A cohort of thirteen age-matched Healthy Control (HC) subjects (9 M, 4 F; age: 39.4 ± 7.2 years) were enrolled as reference population. The healthy subjects met the following inclusion criteria: absence of neurological or musculoskeletal disorders, vestibular diseases, dizziness, long term medications, and bone lesions or joint pathologies of the lower limbs in the year prior to the study.

All the participants had no experience of the procedure; a written informed consent was collected from all participating subjects. The study complied with the principles of the Declaration of Helsinki and the protocol was approved by the Research Ethics Board of the “Bambino Gesù” Children’s Hospital (approval number: 120BCN/VP).

### Equipment

**Fig. 1:**
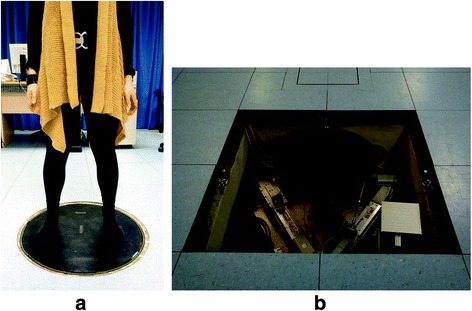
Mechatronic platform with a spherical motion (RotoBiT^3D^) for static and dynamic posturographic tests: **a** subject positioning and **b** view of the mechatronic system. The device for normal operation is hidden in the floor while in the picture the protective layers have been removed to facilitate the mechanism view

Posturography tests were performed using an electrically actuated mechatronic system, the RotoBiT^3D^ [31–34], which permits arbitrary rotations in terms of roll, pitch, and yaw around a fixed point. The mechatronic system, Fig. 1, consists of a moving base connected to three fixed linear electrical actuators by three fixed length arms and six spherical joints. The three actuators are positioned on a fixed platform, which is bolted to an underlying concrete block which provides a high inertia support. The moving base with a diameter of 0.60 m, at the level of the laboratory floor, is also connected to the stationary frame by a spherical joint in order to obtain a pure 3-DOF rotational motion around a fixed point. The three arms are equipped with uni-axial load cells to measure the moment exerted by the subject on the platform [34]. The RotoBiT^3D^ workspace is characterized of about ±10° for roll and pitch angles while yaw angle ranges is ±15°. The phase delay between target and actuated trajectory is 1° and the amplitude error ≤ 1.5% [33]. The robot is integrated in the laboratory floor as shown in Fig. 1. The moving base is equipped with a pressure matrix (MatScan® Pressure Mat, Tekscan®, USA, sampling frequency 100 Hz, 508 x 499.1 mm^2^, number of sensors: 2288) through which the vertical load, the pressure distribution under the feet and position of CoP are then computed.

### Control design

In the perturbed tests, the RotoBiT^3D^ was set in the impedance control mode, which simulated a robot behavior as a 3D spring with a stiffness **K** placed in parallel to a 3D damper with coefficient **C** and an equilibrium angle **γ**_**o**_. The dynamic control system [32], described by the roll-pitch-yaw angles **γ** = (*φ*, *θ*, *ψ*), is subjected to the torque **μ**, computed by means of the uni-axial load cells placed in the three arms of the device:

1 $$ \mathbf{C}\overset{.}{\boldsymbol{\upgamma}}\kern0.5em +\kern0.5em \mathbf{K}\kern0.5em \left({\boldsymbol{\upgamma}}_0\kern0.5em -\kern0.5em \boldsymbol{\upgamma} \right)\kern0.5em =\kern0.5em \boldsymbol{\upmu} $$

The speed control loop is managed through the FPGA board controller (National Instruments, USA) and an additional external speed control is imposed with the following target value:

2 $$ \overset{.}{\boldsymbol{\upgamma}}={\mathbf{C}}^{-1}\left(\boldsymbol{\upmu} -{\boldsymbol{\upmu}}_0\right) $$

where **μ**_**0**_ represents the target torque expressed by:

3 $$ {\boldsymbol{\upmu}}_0=\mathbf{K}\left({\boldsymbol{\upgamma}}_0-\boldsymbol{\upgamma} \right) $$

The criteria here adopted to set **K** and **C** was patient specific. Actually, it was based on the mass and height of the participants and a preliminary static experiment was conducted. A subject of 1.76 m height and a mass of 77.3 kg stood on the platform with a fixed equilibrium roll angle of *φ*_*0*_ = 6° and with a distance between feet of 30 cm. The displacement of CoP was measured during a 20 s equilibrium trial. The CoP showed a maximum displacement from the center of the platform of about 40% of the distance between feet. Thus, a height normalization of the CoP displacement (*dCoP*) for the generic subject’s height (*H*) was evaluated as:

4 $$ dCoP=\left[\frac{H}{176}\right]\cdot 0.4\cdot 30 $$

Then, the stiffness and dumping coefficients patient specific were calculated according to the following equations:

5 $$ \begin{array}{c}\hfill \boldsymbol{K}=\left(\frac{P\cdot dCoP}{\varphi_0}\right)\hfill \\ {}\hfill \boldsymbol{C}=\left(\frac{P\cdot dCoP}{\varphi_0}\right)\cdot \tau \hfill \end{array} $$

Where P is the subject weight and τ, set at 0.5 s, is the time constant that characterizes the model of the mechatronic system implemented in the controller. Finally, the actual base movement is determined from the interaction subject - base support.

### Protocol

Before the experimental protocol, the participants underwent a training session with the RotoBiT^3D^ set in impedance mode, in order to provide subjects with an initial experience, and to familiarize them with the interaction with the moving platform. The training session lasted until participants felt familiar with the equipment and the time length was 1 minute for both HC and patients with MS.

The overall experimental protocol consisted of two sessions that correspond to a Not-Perturbed Test (NPT) and a Perturbed Test (PT). During each session, participants stood barefoot on the platform in standing position, with arms hanging comfortably at their sides and feet placed symmetrically at the center of rotation of the circular moving base. Feet position was marked on the platform, at a distance of about 15 cm from the center of the platform, to assure a consistent position of participants within and across trial blocks. In the eyes open trials (EO), subjects were instructed to look straight ahead and to not gaze at any specific target. In eyes closed trials (EC), the visual feedback was denied to the subjects by providing them with eye masks; participants were also instructed to face forward as if looking straight ahead. In the NPTs, the moving base was kept still in the horizontal position while in the PTs the moving base was controlled in the impedance mode by imposing the equilibrium angle **γ**_**o**_ equal to 0°.

Participants were instructed to maintain equilibrium, restricting their response strategy to a feet-in-place response, unless a fall was imminent; consequently, subjects were free to move body segments to compensate for their instability. Participants were tested individually to avoid any mutual influence. In order to reduce the risk of falls and to minimize interference from external support, a trainer stayed close alongside or behind the participant. The trial did not start until subjects declared themselves ready to begin; moreover a verbal warning was given about five seconds before the trial started. Each trial lasted 30 seconds and was repeated three times, for a total of twelve trials, i.e., 3 trials for each of the following: NPT (eyes open and closed) and PT (eyes open and closed). The order of trials was randomized but it was the same for each subject. Between the trials a time interval of at least 30 s was set, during which participants were free to move on the still platform. The whole experimental protocol lasted not more than 20 min per participant.

### Data analysis

Data were analyzed and the indices were computed by using Matlab® (2012b, MathWorks, USA). All statistical analysis was conducted by using Stata 9.2 (Stata-Corp, College Station TX).

### Indices

Studies on the quantification of postural performance have identified a multitude of indices representative of the individual’s capacity in maintaining postural control, used to investigate the effect of age, diseases, or therapeutic interventions. Among all the feasible measures proposed in the literature, we selected the following: (i) 95% Confidence Ellipse Area (CEA), that is the ellipse that contains at least the 95% of the CoP trajectory [38]; (ii) the Sway Path length (SP) that is the length of CoP trajectory [13]; (iii) the root mean square of CoP displacement in the anteroposterior direction (D_ap_) and in the mediolateral direction (D_ml_) [39]; and, finally, (iv) the Romberg of the CEA and SP evaluated as the ratio between EC and EO indices (R_CEA_ and R_SP_) [40–42]. Specifically, an increase of CEA or SP indicates a lower postural control. The highest value between D_ap_ and D_ml_ identifies the preferred direction of the postural adjustments performed by the subject. The influence of the visual system on the balance capability can be evaluated by means of R_CEA_ and R_SP_ values. Indeed, higher R_CEA_ and R_SP_ values indicate a higher influence of the visual system on the postural control.

### Statistical analysis

All data were tested for normality with the Shapiro-Wilk test. Reliability of parameters for both NPT and PT was analyzed using the Intraclass Correlation Coefficient (ICC) with an ICC(2,1) model. Reliability was classified as excellent (ICC ≥ 0.75), good (0.60 ≤ ICC < 0.75), fair (0.40 ≤ ICC < 0.60), and poor (0.00 ≤ ICC < 0.40) [43]. Comparisons between HC and MS patients were performed by unpaired Student’s *t*-Test, with Welch’s correction when the variances were not equal, or by Mann-Whitney test, as appropriate. Paired *t*-test analysis was performed to compare subjects’ performance with and without visual control. One-way analysis of variance (ANOVA) model was used to compare the Romberg indices across groups in order to assess the effects of visual inputs on patients with and without sensitive impairments (sensory EDSS subscore 0 and ≥ 1 are referred as S0 and S1, respectively). The one-way ANOVA test was also used to compare posturography indices among groups stratified according to the presence of cerebellar impairment (cerebellar EDSS subscore 0 and ≥ 1 are referred as C0 and C1, respectively). Receiver Operating Characteristic (ROC) curve analyses were used to estimate the sensitivity of each index in discriminating HC from MS: an area of 100% represents a perfect discrimination, while an area of 50% represents a worthless model [44]. Continuous data are reported as mean ± Standard Deviation (SD). For all tests statistical significance was set at 0.05. All tests should be understood as exploratory data analysis as no prior power calculation and subsequent corrections for multiple testing were applied.

## Results

### Test-retest reliability

The ICC values for CEA, SP, D_ap_, and D_ml_ evaluated for EC and EO conditions and in HC and MS subjects are reported in Table 2. From an overall analysis, the ICC values ranged from good (0.60) to excellent (0.95) reliability for both groups when performed both NPT and PT sessions; a fair (0.55) reliability was found only for D_ap_ of HC group in EO condition during PTs.

**Table 2:** Values of Intraclass Correlation Coefficient (ICC) for the postural indices in healthy subjects HC and patients with MS in eyes open (EO) and eyes closed (EC) conditions during Not-perturbed Test (NPT) and Perturbed Test (PT)

		ICC			
		NPT		PT	
		HC	MS	HC	MS
EO	CEA [mm^2^]	0.80	0.70	0.72	0.73
	SP [mm]	0.80	0.81	0.69	0.67
	D_ap_ [mm]	0.60	0.66	0.55	0.61
	D_ml_ [mm]	0.73	0.91	0.79	0.75
EC	CEA [mm^2^]	0.76	0.86	0.75	0.84
	SP [mm]	0.72	0.95	0.81	0.89
	D_ap_ [mm]	0.75	0.94	0.61	0.65
	D_ml_ [mm]	0.89	0.95	0.67	0.63

*CEA* confidence ellipse area, *SP* sway path, *D*_*ap*_ root mean square of CoP displacement in the anteroposterior direction, *D*_*ml*_ root mean square of CoP displacement in the mediolateral direction

### Comparison between not-perturbed and perturbed tests

**Fig. 2:**
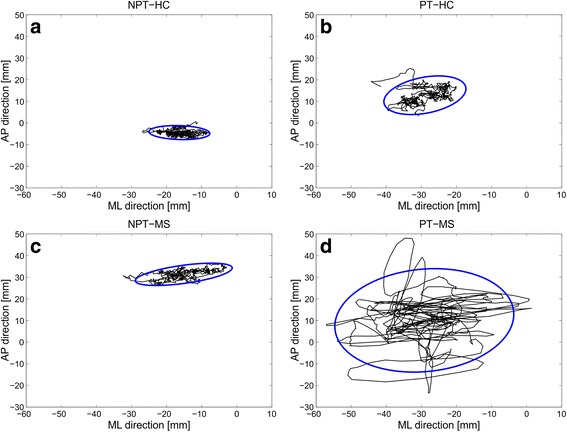
CoP displacements (*black lines*) and Confidence Ellipse Areas (*blue lines*) of a healthy subject **a** and **b** and a patient with MS **c** and **d** recorded during Not-Perturbed Test (NPT) **a** and **c** and Perturbed Test (PT) **b** and **d**

The statokinesigrams of a healthy subject and a subject with MS, assumed as paradigmatic behaviors, are reported in Fig. 2 for NPT and PT.

Table 3 shows the mean and SD values of CEA, SP, D_ap_, and D_ml_ for the two groups. No significant differences were found between HC and MS for all indices when subjects performed the NPT session, irrespectively of the EO or EC condition. During the PT session, instead, significant differences between MS and HC emerged for CEA, SP and D_ap_ in EO condition and for CEA and D_ap_ in EC one.

**Table 3:** Mean and SD values for the postural indices in healthy subjects HC and patients with MS in eyes open (EO) and eyes closed (EC) conditions during Not-perturbed Test (NPT) and Perturbed Test (PT); * represents significant differences between Healthy Control (HC) and subjects with Multiple Sclerosis (MS)

		NPT			PT		
		HC	MS	*p*	HC	MS	*p*
EO	CEA [mm^2^]	85 ± 34	133 ± 159	0.30	656 ± 419	1633 ± 1428	0.02*
	SP [mm]	487 ± 165	459 ± 111	0.58	801 ± 237	1058 ± 378	0.04*
	D_ap_ [mm]	3 ± 1	4 ± 2	0.32	6 ± 2	8 ± 3	0.04*
	D_ml_ [mm]	44 ± 27	31 ± 18	0.13	24 ± 16	25 ± 13	0.79
EC	CEA [mm^2^]	235 ± 309	108 ± 50	0.15	3553 ± 1770	7506 ± 5418	0.02*
	SP [mm]	514 ± 173	589 ± 231	0.33	1816 ± 586	2362 ± 1142	0.13
	D_ap_ [mm]	4 ± 2	6 ± 3	0.07	14 ± 4	18 ± 5	0.02*
	D_ml_ [mm]	43 ± 28	32 ± 18	0.20	30 ± 17	29 ± 10	0.83

*CEA* confidence ellipse area, *SP* sway path, *D*_*ap*_ root mean square of CoP displacement in the anteroposterior direction, *D*_*ml*_ root mean square of CoP displacement in the mediolateral direction

The high discriminating ability of PT session between mildly disabled MS patients and HC was confirmed by means of the ROC analysis (Table 4) with CEA and D_ap_ showing values of at least 70% in EO condition, thus indicating a fair goodness in the discrimination [34]. The EC trials further increased the discriminating ability of CEA, determining the best discriminating power (76.4%). As expected, all NPT trials had lower discriminating power compared to PT, and none reached the value of 70.0%, which can be considered the lowest limit for a fair good discrimination.

**Table 4:** ROC analysis area values obtained in Not-perturbed Test (NPT) and Perturbed Test (PT) for the postural indices in eyes open (EO) and eyes closed (EC) conditions

		ROC [%]	
		NPT	PT
EO	CEA	47.9	71.0
	SP	48.8	69.6
	D_ap_	60.6	70.5
	D_ml_	36.2	54.3
EC	CEA	56.1	76.4
	SP	61.5	62.4
	D_ap_	69.2	73.7
	D_ml_	41.1	47.5

### Perturbed tests are sensitive to detect cerebellar disturbances on balance performance in subjects with MS

**Fig. 3:**
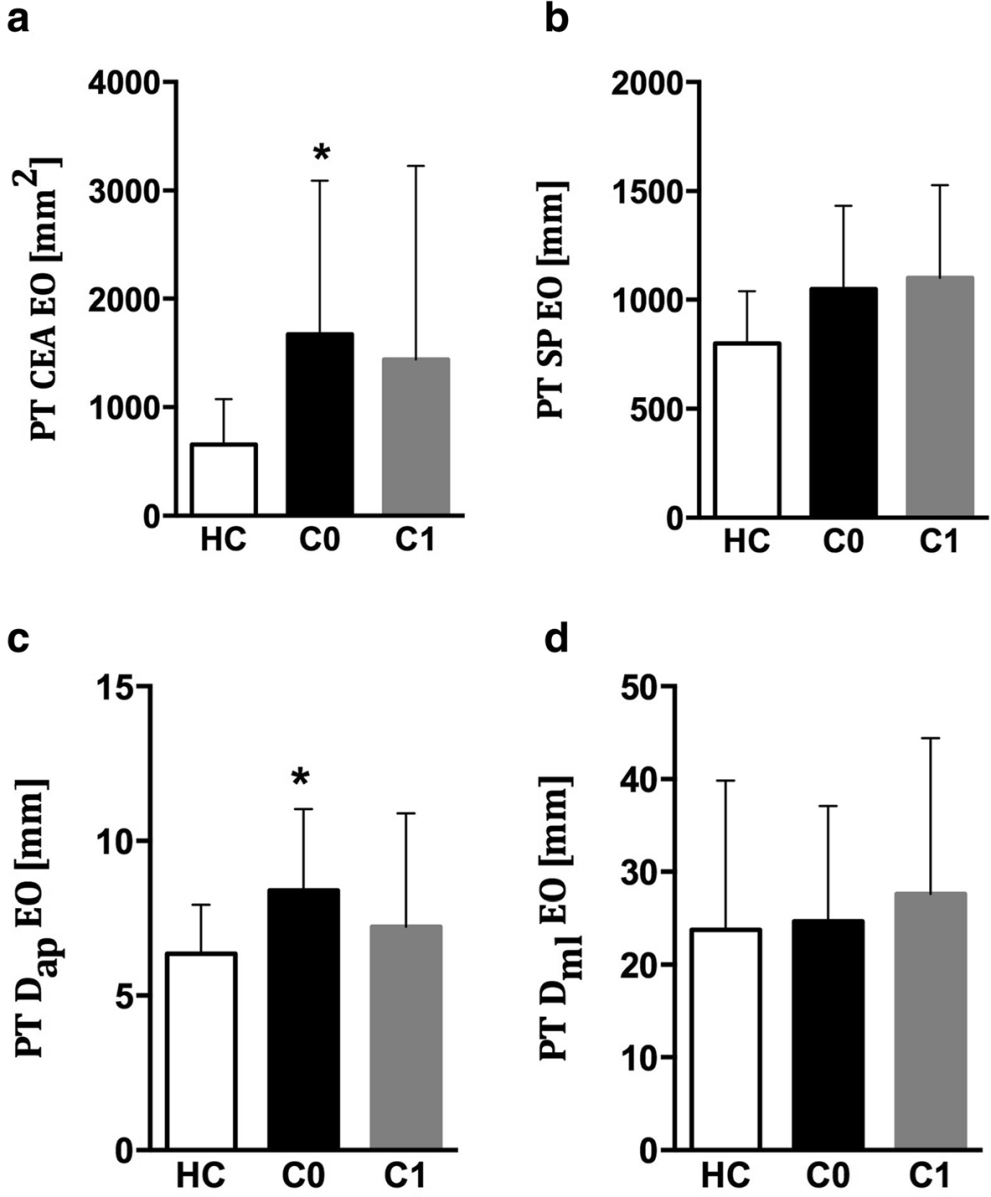
Confidence Ellipse Area (CEA), Sway Path (SW), anteroposterior displacement of CoP (D_ap_), and mediolateral displacements (D_ml_) evaluated for Healthy Controls (HC), and MS patients with (C1) and without (C0) cerebellar impairment

In order to further investigate whether the sensitivity of the PT postural variables could help to individuate balance disturbances before any clinical evidence, MS patients were categorized according to the absence or the presence of cerebellar impairment at clinical evaluation (referred to as C0 and C1, respectively) and, consequently, the postural indices were computed in accordance to the two subgroups, as shown in Fig. 3. Our results show that even patients without any cerebellar impairment (C0) presented significantly different postural indices during PT trials, respect to HC (CEA *p* = 0.03, F = 2.7; D_ap_: *p* = 0.03, F = 2.5; SP: *p* = 0.06, F = 2.2; D_ml:_ *p* = 0.99, F = 0.08). No correlation was revealed between any of the aforementioned variables and global disability score (EDSS), or motor disability (EDSS subscore).

### Not-perturbed tests are sensitive to detect sensory disturbances on balance performance in MS

**Fig. 4:**
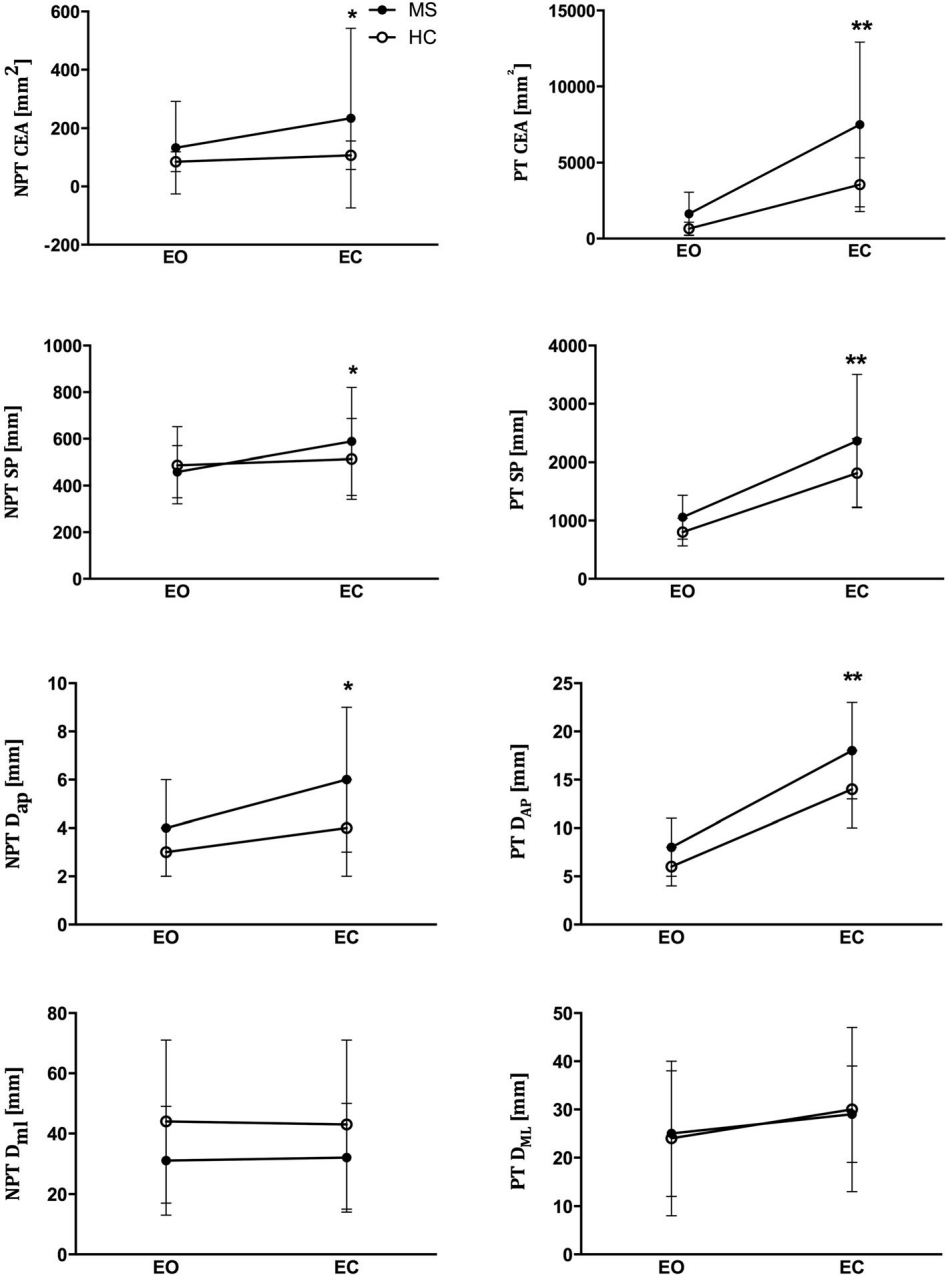
Effects of visual control on balance. Confidence Ellipse Area (CEA), Sway Path (SW), anteroposterior displacement of CoP (D_ap_), and mediolateral displacements (D_ml_) evaluated during NPT and PT sessions with eyes closed (EC) and eyes open (EO) conditions. *: *p* < 0.05 respect to EO condition only for MS subjects. **: *p* < 0.05 respect to EO condition for both HC and MS subjects

Further analyses were performed to investigate the role of sensory disturbances on balance performance. As balance control relies on proprioception after the withdrawal of visual feedback, we analyzed the worsening of postural variables during EC trials as a measure of sensory function, see Fig. 4.

No significant worsening occurred in HC during NPT performed with eyes closed (HC: *p* > 0.05 for each comparison, Fig. 4). Conversely, all indices, with the exception of D_ml_, presented a significant increase in MS subjects. (MS: CEA: *p* = 0.03, SP: *p* = 0.003, D_ap_: *p* < 0.001, D_ml_: *p* = 0.39, Fig. 4). The same analysis performed for PT revealed a significant worsening in CEA, SP and D_ap_ both in HC and MS (*p* < 0.001 for each comparison for both groups, Fig. 4), while D_ml_ was not significantly affected (MS: *p* = 0.16, HC: *p* = 0.09).

**Fig. 5:**
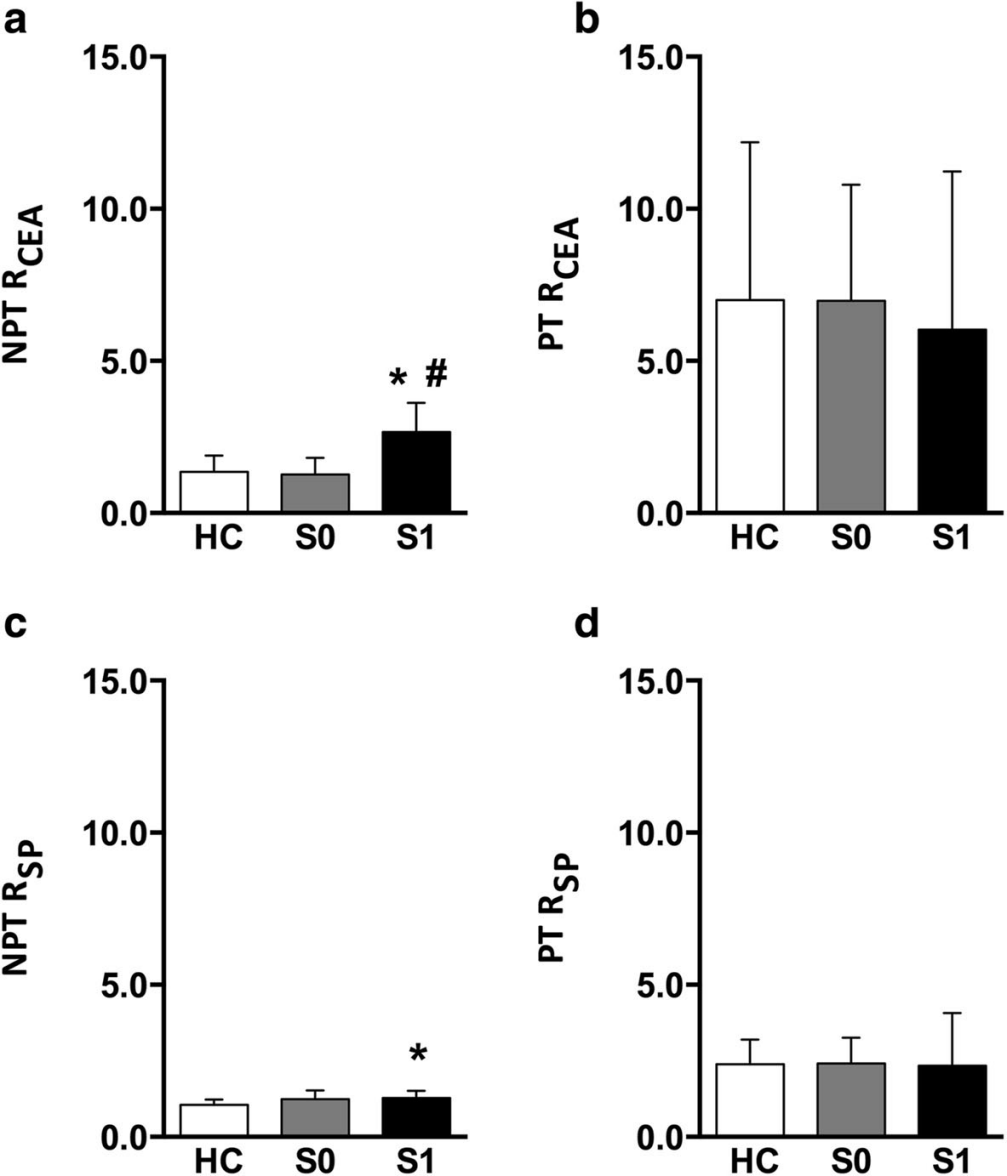
Romberg Indices of CEA (R_CEA_) and SP (R_SP_) in NPT and PT sessions evaluated for Healthy Controls (HC), and MS patients with (S1) and without (S0) sensory impairment. *:*p* <0.05 respect to HC; #:*p* < 0.05 respect to S0

Romberg indices of CEA and SP were considered to quantify the impact of loss of visual feedback on balance performance. The Romberg indices for PT and NPT are reported in Table 5. Our results show that, while R_SP_ gathered during NPT (*p* = 0.02) was higher in MS patients respect to HC, R_CEA_ was comparable between the two groups (*p* = 0.12). Moreover, the Romberg indices were categorized for patients with and without sensory impairments at clinical evaluation (referred to as S1 and S0, respectively), see Fig. 5. S1 patients showed higher R_SP_ respect to HC and higher R_CEA_ respect to both S0 and HC (*p* < 0.05, Fig. 5a and c). Lastly, no differences were found among subgroups in PT (*p* > 0.99 for each comparison for both CEA and SP, Fig. 5b and d).

**Table 5:** Mean and SD values for the Romberg indices obtained in Not-perturbed Test (NPT) and Perturbed Test (PT) in healthy subjects HC and patients with MS; * represents significant differences between HC and MS

	NPT			PT		
	HC	MS	*p*	HC	MS	*p*
R_CEA_	1.3 ± 0.5	1.8 ± 1.0	0.12	7.0 ± 5.2	6.6 ± 4.3	0.81
R_SP_	1.1 ± 0.2	1.3 ± 0.3	0.02*	2.4 ± 0.8	2.4 ± 1.2	0.99

## Discussions

MS is a chronic progressive neurologic disease in which an impaired central integration of visual, vestibular and somatosensory input may lead to postural control disorders and increased risk of falls [2, 3]. As a consequence, MS patients may experience difficulties in maintaining equilibrium in not-perturbed conditions [2, 15] and mostly in perturbed ones [10, 27, 45, 46].

In this study we compared the sensitivity of postural indices in detecting balance alterations in a group of MS patients with low disability and without history of falls. The main finding of the present study is that the perturbed posturography showed a good sensitivity in detecting postural control alterations in patients with minimum or even absent clinical evidence of balance disturbances, while static postural indices failed to highlight significant differences compared to healthy subjects.

The lack of statistical differences between patients and control subjects, on the performance indices computed for NPT, apparently contradicts the results shown by Prosperini et al. [15]. It could be justified considering the relatively small sample size and the different level of disability of patients involved in the two studies. In fact, we included MS patients with lower disability and no history of falls, thus probably requiring more challenging balance trials to reveal subtle difficulties in maintenance of upright stance.

On the contrary, the indices CEA, SP and D_ap_ computed during PT showed a good sensitivity in discriminating mildly affected MS patients, even when the analysis was restricted to patients with no clinical signs of cerebellar impairment for CEA and D_ap_.

Consequently, our findings allow us to confirm that the PTs are more sensitive than NPTs to discriminate MS subjects, as also reported by Fjeldstad et al. [27] and particularly that the PTs are able to discriminate even MS subjects with no cerebellar impairments from healthy subjects. This ability is due to the more challenging trials consisting in multi direction perturbations imposed by our experimental setup that is consequently able to reveal even subtle balance difficulties. Our results are in line with previous findings that balance control during stance is less discriminating than during gait in minimally impaired MS patients [21] and that only more challenging stance trials are able to reveal differences among these patients respect to healthy subjects [17].

A further noteworthy finding revealed by using a complex perturbation is that a preferential direction in COP displacement has been identified in our sample of MS patients: specifically, during PT, patients increased their body sway along the anteroposterior direction, as reported by the higher values of D_ap_, respect to HC. These findings are consistent with the greater CoP displacement in the AP than ML direction found in women with MS. As lateral balance control derives from the weighting and unweighting of each limb [47], the increased lateral sway might result from asymmetric weight load between left and right leg during the execution of the required task. Higher EDSS scores were correlated with reduced lateral balance control [48]. In our sample, no subjects with high EDSS and/or marked asymmetry of limb weakness were included; this could therefore account for not having recorded significant differences in D_ml_ and association with global disability. Our observation of increased anteroposterior deviation suggests different balance strategies in MS respect to other neurological diseases, such as Parkinson disease, in which a predominant imbalance in mediolateral direction was instead described [49]. Thus, the proposed robotic system, which is capable to give complex perturbations not limited to a single rotation axis, represents a sensitive system in the discrimination of preferential movement directions. The possibility to investigate correlation between disease and direction of balance adjustments can represent an important improvement with respect to the other balance tests that have been already proposed in the literature, such as SOT or tests based on translational perturbations.

Other novel and relevant information derive from tests performed without visual control. Our results show that, in EC NPTs, HC were able to keep balance thanks to compensatory strategies connected with proprioceptive feedback; conversely, a worse balance performance was registered in MS subjects, with a significant increase of Romberg indices, due to a less efficient proprioceptive feedback. On the contrary, the combination of two balance-challenging conditions, like perturbed posturography and closure of the eyes, leads to a worsening of balance indices in both patients and controls, suggesting a greater dependence of these parameters on visual feedback, with a ceiling effect of the capabilities of postural adjustments even in healthy subjects. For this reason, while the more sensitive PTs are needed to detect balance impairment when no other balance challenge is required, the deprivation of visual feedback might reduce PTs specificity because of an excessive complexity of the motor task. Indeed, the differences between groups in Romberg Indices recorded during NPT, were lost in PT. In particular, we found a higher R_SP_ for MS than healthy subjects in NPT, indicating that MS patients showed a compromised balance control after the withdrawal of visual stimuli; analogous reports were recently provided [50]. This result, however, was not mirrored by an analogous R_CEA_ difference between the two groups. Thus, simple perturbations based on movements of the base support in only one direction could be more useful than complex perturbations in the highlighting differences between HC and MS when balance control strategies in absence of visual feedback are studied.

In order to gain deeper insight into this finding, we performed further analysis to assess the influence of proprioceptive disturbances on Romberg Indices in MS patients. We therefore stratified MS subjects in two subgroups according to the presence of clinically evident sensitive impairments. We found that the sensitive impaired MS group showed significant higher values of R_CEA_ relatively to both healthy subjects and to sensitively preserved MS patients. Conversely, R_SP_ was less influenced by the presence of a clinically evident sensitive impairment. We can therefore postulate that CEA is more closely associated with proprioceptive afference contribution to balance maintenance, with R_CEA_ representing a reliable index of patients’ sensitive impairments.

Hence, from an overall analysis of the obtained results we can conclude that the use of the novel perturbed test based on a 3-DOF mechatronic device represents a new and sensible tool for investigating early balance disturbances and monitoring disability course among subjects with MS.

Limitations of the present study mainly concern the small sample size, which could reduce the statistical power of our findings and the chance to stratify patients according to clinical conditions in further detail. Moreover, even if no statistically significant difference was found between HC and MS group with respect to age and genre, controls were younger than patients; this could have partially influenced motor performance, as younger age is generally associated to better balance. Further studies on larger and balanced samples are needed to widely clarify the role of dynamic stabilometric platform in characterizing balance performance in minimally impaired MS subjects.

## Conclusion

Our findings confirm that postural indices evaluated in perturbed conditions show higher sensitivity respect to common static tests in discriminating and quantifying postural performance in MS patients. The higher values of displacement in the anteroposterior direction of the patients in dynamic condition highlighted a poor control of stability in the sagittal plane in MS disease. Instead, static tests are more reliable to detect the effects of sensory disturbances on balance performance.

## Abbreviations

C0: Multiple sclerosis group with no cerebellar impairment
C1: Multiple sclerosis group with cerebellar impairment
CEA: Confidence ellipse area
CNS: Central nervous system
CoP: Center of pressure
Dap: Root mean square of CoP displacement in the anteroposterior direction
D_ml_: Root mean square of CoP displacement in the mediolateral direction
EC: Eyes closed
EDSS: Expanded disability status scale
EO: Eyes open
HC: Healthy control
MS: Multiple sclerosis
NPT: Not-perturbed test
PT: Perturbed test
R: Romberg ratio
ROC: Receiver operating characteristic
S0: Multiple sclerosis group without sensitive impairment
S1: Multiple sclerosis group with sensitive impairment
SD: Standard deviation
SOT: Sensory organization test
SP: Sway path
